# Impact of Age on the Efficacy of Immune Checkpoint Inhibitor-Based Combination Therapy for Non-small-Cell Lung Cancer: A Systematic Review and Meta-Analysis

**DOI:** 10.3389/fonc.2020.01671

**Published:** 2020-09-23

**Authors:** Xin Yan, Xuan Tian, Zhiqiang Wu, Weidong Han

**Affiliations:** ^1^Department of Bio-Therapeutic, The First Medical Center, Chinese People's Liberation Army General Hospital, Beijing, China; ^2^School of Medicine, Nankai University, Tianjin, China

**Keywords:** immune checkpoint inhibitor, age, meta-analysis, combination, non-small-cell lung cancer

## Abstract

**Background:** Despite the acknowledged benefits of immune checkpoint inhibitor (ICI)-based combination therapy (either with other checkpoint inhibitors, chemotherapy, targeted therapy, or radiotherapy), little is known about the impact of age on the efficacy of ICI -based combination therapy in non-small-cell lung cancer (NSCLC) patients. We conducted a systematic review and meta-analysis to investigate the differences in the benefits of ICI-based combination therapy for NSCLC by age (cut-off age, 65 years).

**Methods:** We systematically searched randomized controlled trials (RCTs) of ICI plus other therapies including other ICIs, chemotherapies, targeted therapies, or radiotherapies, in the PubMed, Embase, and Cochrane databases with available hazard ratios (HRs) and 95% confidence intervals (CIs) for death and disease progression according to patient age. The search deadline was May 25, 2020. First, we calculated the pooled HRs of younger and older patients based on the HRs from each trial. Second, we assessed the pooled ratio of HRs reported in older patients to the HRs reported in younger patients for progression or death by the random-effects model. An estimated pooled HR ratio was lower than 1 indicating a better effect in older patients and higher than 1 indicating a better effect in younger patients.

**Results:** A total of 10 eligible RCTs were included in our meta-analysis. The pooled HR for overall survival (OS) comparing ICI combined with other therapies to non-ICI regimens was 0.67 (95%CI 0.58–0.78) for younger patients and 0.79 (95%CI 0.70–0.90) for older patients. The pooled HRs ratio for OS reported in older patients compared to younger patients was 1.16 (95%CI 0.99–1.34), indicating no statistically significant difference between younger and older patients. Consistent with the findings related to OS, the analysis also demonstrated that ICI-based immunotherapy could significantly prolong progression-free survival (PFS) in younger and older patients (HR = 0.55; 95% CI 0.47–0.66, and HR = 0.64; 95% CI 0.57–0.71). The same results could also be observed in the pooled HRs ratio for PFS (HR = 1.15, 95%CI 0.91–1.46) indicating comparable efficacy of ICI-based combination therapy in younger and older patients with NSCLC.

**Conclusion:** ICI-based combination therapy vs. non-ICI treatment had comparable efficacy in younger and older NSCLC patients with a cut-off age of 65 years.

## Introduction

The emergence of immune checkpoint inhibitors (ICIs) has transformed the paradigm of clinical management of lung cancer (1). Nevertheless, only ~20% of patients with advanced lung cancer can benefit from monotherapy with ICI (2). More recently, ICI plus other therapies like other ICI, chemotherapy, radiotherapy, and targeted therapy, have been shown to synergistically promote the efficacy of ICI monotherapy in non–small-cell lung cancer (NSCLC) patients, which has been confirmed in several randomized controlled trials (RCTs) and systematic reviews (3–11).

Cancer mainly occurs in older patients, and age is also associated with a poor prognosis for cancer (12). Compared with younger people, the immune system of older people can undergo a remodeling process during aging, called immunosenescence, which involves a diminishing ability to resist tumors and a decline in various immune cell functions (13). Immunosenescence can promote a reduction in the number of CD8^+^ T cells, including a decrease of T cell receptor diversity and proliferative capacity (14, 15). In addition, immunosenescence can cause functional defects, such as decreased expression of CD28 (16), upregulation of Tim-3 and programmed cell death protein 1(PD-1) (17, 18), decreased cytokine production and IL-2 signaling, and reduced secretion of perforin and granzyme (12, 19–21). Based on the preclinical data mentioned above, it is speculated that older patients obtain limited benefits from ICI treatment compared to younger patients. However, some studies have reported that the benefit of ICI treatment is independent of age (21–23).

Generally, due to a decline of physical and physiological functions, many older patients cannot tolerate ICI combination immunotherapy, chemotherapy, radiotherapy, or targeted therapy. Some reports have also demonstrated that ICI combination immunotherapy, chemotherapy, radiotherapy, and/or targeted therapy is less effective in older people than in younger people (5, 24–27).

It has been indicated that other therapies can synergistically enhance the anti-tumor effectiveness of ICI in the overall population. Due to the fact that the physical condition of elderly patients is generally inferior to that of younger people, older people often do not meet the rigorous selection criteria of many clinical trials, leading to insufficient data from older people in RCTs (28–32). Moreover, due to the complexity of age, as it relates to antitumor immunity, it is unclear whether ICI-based combination therapy has a better effect than non-ICI therapy in older patients. As a result, we performed a systematic review and meta-analysis to compare the efficacy of ICI- based combination therapy based on age, examining younger vs. those older than 65 years among NSCLC patients.

## Methods

### Search Strategy and Study Selection

This meta-analysis was conducted according to the Preferred Reporting Items for Meta-analyses (PRISMA) statement (33). Eligible randomized controlled trials comparing ICI-based combination therapy with non-ICI regimens were identified from the PubMed, Embase, and Cochrane databases through May 25, 2020.

YX and TX searched the databases separately. The search keywords were immune checkpoint inhibitor, programmed cell death-ligand 1 (PD-L1), PD-1, cytotoxic T lymphocyte-associated antigen-4 (CTLA-4), non-small-cell lung cancer, and randomized controlled trial. We also reviewed the references of all final selection studies (Supplementary Table 1).

The following inclusion criteria were used by PICOS structure: (1) Population: NSCLC patients. (2) Intervention: PD-1 or PD-L1 or CTLA-4 inhibitor plus other therapies including other ICIs, chemotherapies, targeted therapies, and radiotherapies. (3) Control: non-ICI therapy. (4) Outcomes: available hazard ratios (HRs) and 95% confidence intervals (CIs) for overall survival (OS) and/or progression-free survival (PFS) with information on patient age. (5) Study: Phase II or III RCTs. (6) Published in English. We excluded single-arm studies. Regarding duplicate studies, only those with the most complete and up-to-date data were included.

### Data Extraction

YX and TX extracted data separately using a predefined information list. All of the disagreements were resolved by consensus with all investigators. Study characteristics were extracted, including study name, National Clinical Trial (NCT) number, first author, year of publication, treatment agents, line of therapy, histology of lung cancer, baseline demographic characteristics, and HRs stratified by age (younger vs. ≥ 65 years) for PFS and/or OS.

When studies with duplicate data existed, we included data from the most complete and recent study. A risk-of-bias assessment was performed with the Cochrane Collaboration tool for assessing the risk of bias (34).

### Statistical Analysis

The primary objective was to evaluate the efficacy of ICI-based combination therapy in younger (<65 years) and older (≥65 years) patients, measured as a ratio of the HRs for progression or death between older patients and younger patients.

We extracted the HRs and 95% CIs for death or progression in the intervention arm and control arm from each trial, for younger and older patients. The HRs for patients 65–70 and ≥70 years reported in articles were estimated based on the random-effects model, and the estimated HR (≥65 years) was then included in our meta-analysis.

We used the Q test and *I*^2^ values to determine and quantify heterogeneity between studies (35). HRs and CIs were converted to log HRs and corresponding variances. Due to the inherent clinical heterogeneity of the data, we used random-effects models for all meta-analyses. First, we calculated the pooled HR of younger and older patients based on HR from each trial. Second, we compared the pooled ratio of HRs reported in older patients to the HRs in younger patients for progression or death by the random-effects model (36, 37). If the pooled HR was <1, it means that older patients benefited more from ICI-based combination therapy compared to non-ICI regimens than younger patients. In contrast, if the pooled HR was >1, it means that the younger patients benefitted more.

Subgroup analysis was conducted to detect the underlying source of heterogeneity in terms of the type of ICIs and treatment strategy. Sensitivity analysis was performed to assess the stability of the overall estimate by moving one trial at a time. Because the number of included studies was not more than 10, we did not assess publication bias. All reported *P-*values are 2 sides, and *P* ≤ 0.05 was considered statistically significant. All computations were conducted by StataMP (version 14).

## Results

### Search Results and Patient Characteristics

**Figure 1:**
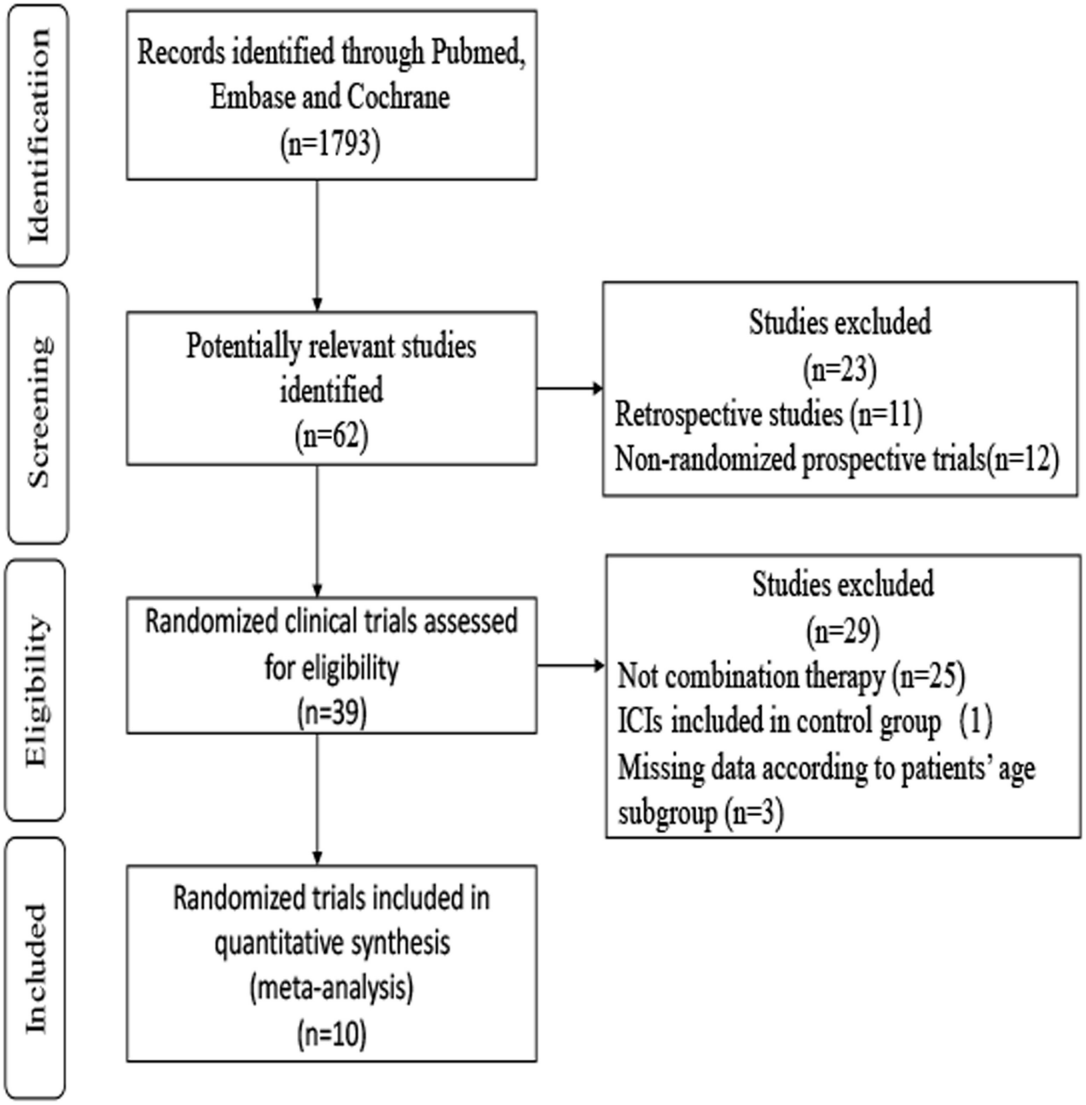
Flowchart depicting the RCTs selection process.

A total of 1,793 records were identified through searches of PubMed, Embase, and Cochrane. After screening the abstracts and reviewing the full texts, the final meta-analysis included 10 trials involving 6,469 patients (4, 5, 7–11, 38–42). A flowchart depicting the RCT selection process is shown in Figure 1.

The 10 included studies were RCTs of ICI-based combination therapy vs. non-ICI regimens. The main characteristics of the 10 RCTs are summarized in Table 1. All studies included HRs and 95% CIs based on age subgroups for OS or/and PFS. Among the 10 studies, eight studies assessed the efficacy of combination therapy for OS, while seven studies assessed the efficacy of combination therapy for PFS. Five trials investigated PD-L1 inhibitors (atezolizumab and durvalumab), four investigated PD-1 inhibitors (pembrolizumab and nivolumab), and two investigated CTLA-4 inhibitors (ipilimumab). Six trials researched ICI combined with chemotherapy, two trials researched ICI combination therapy, one trial researched ICI plus targeted therapy, and one trial researched ICI plus radiotherapy. The age range of patients was 18–90 years, and 3 142 (48.6%) participants were 65 years or older. The assessment of the risk of bias is provided in Supplementary Table 2. There was a low risk of bias for random sequence generation and selection in all the studies. However, the Impower130 study (4) was an open-label study, and the data from Impower131 and Impower 132 study (8, 39) could only be obtained from the abstract and the presentation slides, which may increase the risk of bias. Generally, the quality of these trials was satisfactory.

**Table 1:** Characteristics of patients in the included trials.

	**Study name**	**Phase**	**Malignancy**	**Lines**	**Treatment (No.)**	**Age (y) median (range)**	**<65y (*N*o.)**	**≥65y (*N*o.)**	**OS**			**PFS**		
									**Overall HR (95% CI)**	**HR (95%CI) <65 y**	**HR [(95%CI)] ≥65 y**	**Overall HR (95% CI)**	**HR (95%CI) <65 y**	**HR [(95%CI)] ≥65 y**
West et al. (4) (2019)	IMpower 130	III	NSCLC	1	ATE+ CBDCA +nTxl(451) CBDCA +nTxl(228)	64 (18–86) 65 (38–85)	341	338	0.79 (0.64–0.98)	0·79 (0.58–1.08)	0.78 (0.58–1.05)	0.64 (0.54–0.77)	0.64 (0.5–0.82)	0.64 (0.5–0.82)
Hellmann et al. (5) (2019)	CheckMate 227b	III	NSCLC	1	NIV+IPI (396) CDDP/CBDCA+Pem (397)	64 (26–84) 64 (27–85)	406	387	0.79 (0.65–0.96)	0.70 (0.55–0.89)	0.91 (0.70–1.13)^#^	NR	NR	NR
Jotte et al. (8) (2018)	IMpower 131	III	NSCLC	1	ATE+ CBDCA+nTxl(343) CBDCA+ nTxl(340)	65 (23–83) 65 (38–86)	326	357	NR	NR	NR	0.71 (0.60–0.85)	0.77 (0.61–0.99)	0.62 (0.49–0.79)^#^
Papadimitrakopoulou et al. (39, 40) (2018)	IMpower 132	III	NSCLC	1	ATE+ CBDCA/CDDP+ Pem(292) CBDCA/CDDP+ Pem(286)	NR NR	320	258	0.81 (0.64–1.03)	0.89 (0.62–1.21)	0.71 (0.50–1.01)	0.60 (0.49–0.72)	0.63 (0.49–0.80)	0.55 (0.42–0.73)
Gandhi et al., (9) (2018)	KEYNOTE 189	III	NSCLC	1	PEM+ CDDP/CBDCA+Pem (410) placebo+ CDDP/CBDCA+Pem(206)	65. (34–84) 63.5(34–84)	312	304	0.49 (0.38–0.64)	0.43 (0.31–0.61)	0.64 (0.43–0.95)	0.52 (0.43–0.64)	0.43 (0.32–0.56)	0.75 (0.55–1.02)
Paz-Ares et al. (38) (2018)	KEYNOTE 407	III	NSCLC	1	PEM+ CBDCA+Txl/nTxl(278) CBDCA+Txl/nTxl(281)	65 (29–87) 65 (36–88)	254	305	0.64 (0.49–0.85)	0.52 (0.34–0.80)	0.74 (0.51–1.07)	0.56 (0.45–0.70)	0.50 (0.37–0.69)	0.63 (0.47–0.84)
Hellmann et al. (41) (2018)	CheckMate 227a	III	NSCLC	1	NIV+IPI (139) CDDP/CBDCA+Pem (160)	64 (41–87) 64 (29–80)	156	143	NR	NR	NR	0.58 (0.41–0.81)	0.51 (0.34–0.77)	0.59 (0.39–0.89)^#^
Antonia et al. (10, 42) (2018)	PACIFIC	III	NSCLC	≥2	DUR+ chemoradiotherapy(476) Placebo+ chemoradiotherapy(237)	64 (31–84) 64 (23–90)	391	322	0.68 (0.47–1.00)	0.62 (0.44–0.86)	0.76 (0.55–1.06)	0.51 (0.41–0.63)	0.43 (0.32–0.57)	0.74 (0.54–1.01)
Socinski et al. (7) (2018)	IMpower 150	III	NSCLC	1	ATE+Bev+CBDCA+Txl (400) Bev+CBDCA+Txl (400)	63 (31–89) 63 (31–90)	441	359	0.78 (0.64–0.96)	0.65 (0.51–0.82)	0.60 (0.41–0.87)^#^	0.62 (0.52–0.74)	NR	NR
Govindan et al. (11) (2017)	CA184-104	III	NSCLC	1	IPI+ CBDCA+Txl(388) Placebo+ CBDCA+Txl(361)	64 (28–84) 64 (28–85)	380	369	0.91 (0.77–1.07)	0.82 (0.64–1.04)	1.01 (0.80–1.28)^#^	0.87 (0.75–1.01)	NR	NR

# *NR (not reported); estimated HR calculated by a random-effects model*.

*OS, overall survival; PFS, progression-free survival; HR, hazard ratio; CI, confidence interval; NSCLC, non-small lung cancer; ATE, atezolizumab;PEM, pembrolizumab; NIV, nivolumab; DUR, durvalumab; CBDCA, carboplatin; Pem, pemetrexed; IPI, ipilimumab; CDDP, cisplatin; Txl, paclitaxel; nTxl, nab-pacltitaxel; Bev, bevacizumab*.

### Pooled HRs in Younger and Older Patients With NSCLC

**Figure 2:**
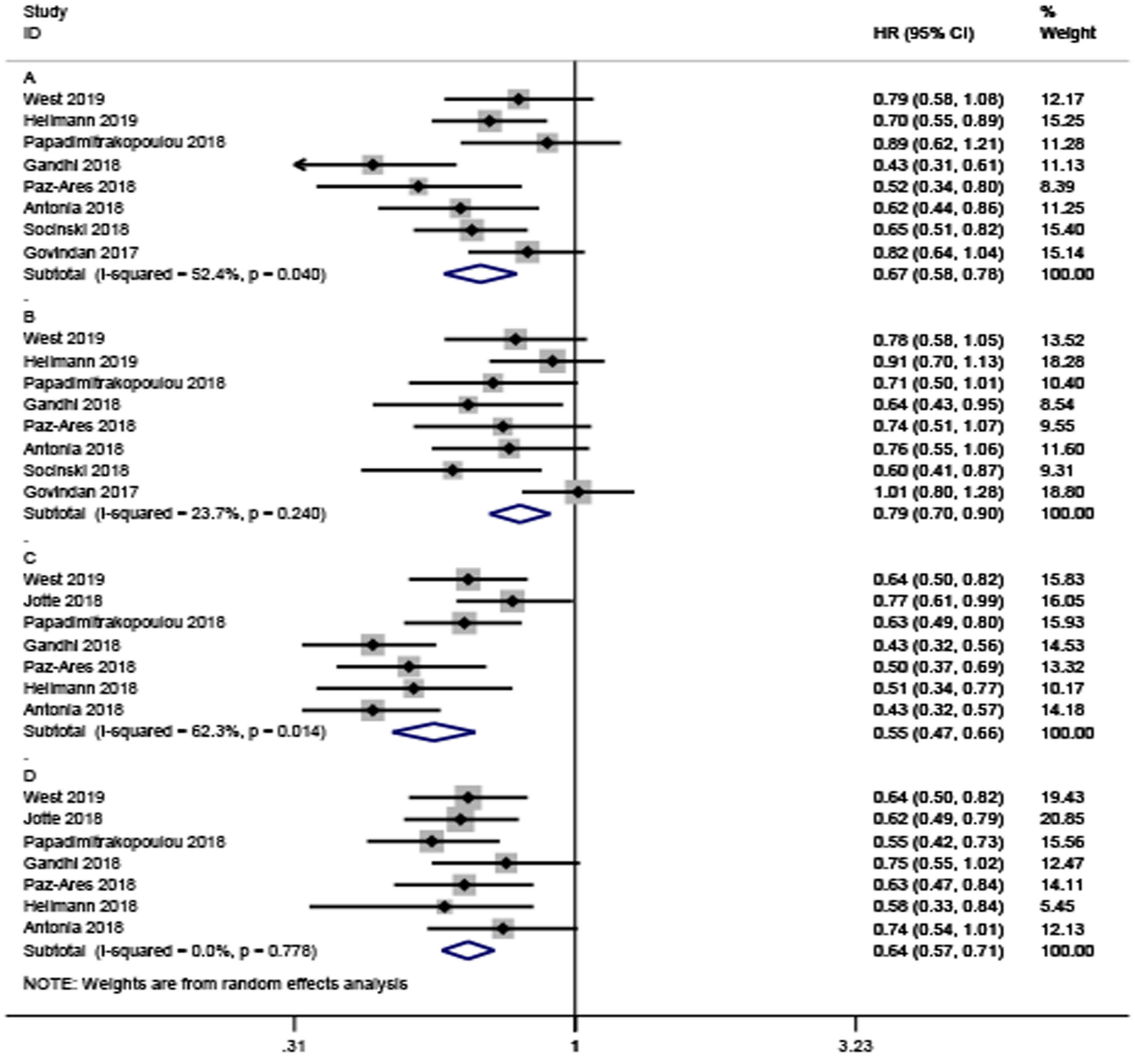
Forest plots of hazard ratios for death and progression by patient age. **(A,B)** Are the hazard ratios for death in younger and older patients, respectively. **(C,D)** Are the hazard ratios for progression in younger and older patients, respectively.

A total of eight trials with 5,487 participants focused on the efficacy of ICI-based combination therapy and provided OS value stratified by age. For younger patients, the pooled HR showed a significant difference between ICI-based combination therapy and non-ICI strategies (HR = 0.67, 95% CI 0.58–0.78). The random-effects model was used to evaluate heterogeneity (*P* = 0.04, *I*^2^ = 52.4%; Figure 2A). Similarly, the combination therapy greatly prolonged OS (HR = 0.79, 95% CI 0.70–0.90) with heterogeneity (*I*^2^ = 23.7%, *P* = 0.24, Figure 2B) found among older patients.

Only seven studies with a total of 4,127 participants investigated PFS. Consistent with OS, the analysis also confirmed that ICI-based combination therapy could significantly prolong PFS in younger and older patients (HR = 0.55; 95% CI, 0.47–0.66, and HR = 0.64; 95% CI 0.57–0.71, respectively; Figures 2C,D). There was significant heterogeneity among younger participants (*I*^2^ = 62.3%, *P* = 0.014), but no significant heterogeneity among older participants (*I*^2^ = 0%, *P* = 0.776).

### Pooled Ratio of HRs in Older Patients to HRs in Younger Patients With NSCLC

The above analysis revealed that both older and younger patients can experience a reduced risk of death or progression as a result of the addition of ICIs. Next, we further evaluated the difference between older and younger patients regarding the efficacy of ICI combined with other therapies for NSCLC treatment.

**Figure 3:**
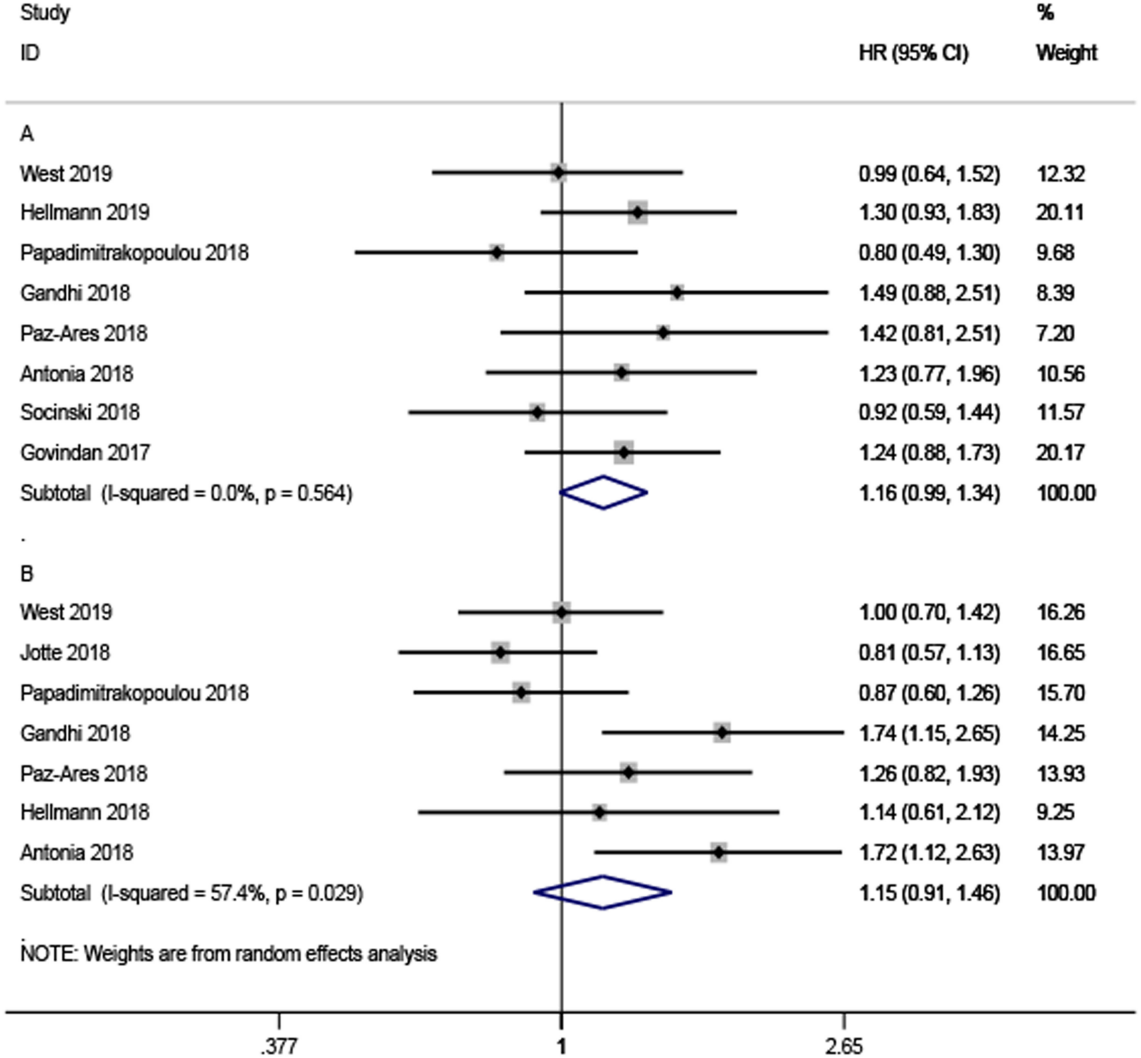
Forest plots of the ratio of hazard ratios in older patients to younger patients for death **(A)** and progression **(B)**.

The pooled ratio of the HRs for OS reported in older NSCLC patients vs. the HRs for OS reported in younger patients in each study was 1.16 (95% CI, 0.99–1.34, Figure 3A) without significant heterogeneity (*I*^2^ = 0%, *P* = 0.564). This indicated a similar OS benefit obtained from ICI-based combination therapy in younger NSCLC patients. The same results could also be observed in the HR ratio for PFS (HR = 1.15, 95% CI, 0.91–1.46, Figure 3B) with significant heterogeneity (*I*^2^ = 57.4%, *P* = 0.029), indicating comparable efficacy of ICI-based combination therapy in younger and older participants with NSCLC.

### Subgroup Analysis by Type of ICI and Treatment Strategy

We conducted a subgroup analysis stratified by type of ICI and treatment strategy (Supplementary Figures 1–8). In all subgroup analyses for OS, the ratios of the HRs reported in older people to the HRs reported in younger people were found to indicate comparable benefits from ICI-based combination therapy with no significant difference. However, subgroup analyses of OS revealed a difference between younger and older people.

In subgroup analysis based on the ICI type, compared with non-ICI treatment, both younger (HR = 0.72, 95% CI 0.61–0.84, *I*^2^ = 10.6%) and older patients (HR = 0.72, 95% CI 0.61–0.84, *I*^2^ = 0%) could obtain prolonged survival through treatment with PD-L1 inhibitor-based combination therapy without significant heterogeneity. Consistent results were obtained for OS-based PD-1 inhibitor combination therapy in younger (HR = 0.46, 95% CI 0.35–0.60, *I*^2^ = 0%) and older patients (HR = 0.69, 95% CI 0.53–0.91, *I*^2^ = 0%). Although both younger and older people can benefit from PD-1/PD-L1 blocker combination therapy in terms of PFS, younger people could benefit more according to the pooled ratio of the HRs of older patients to the HRs of younger patients treated with PD-1 inhibitor combination therapy (HR = 1.49, 95% CI 1.08–2.04, *I*^2^ = 10.7%).

In subgroup analysis based on the treatment strategy, the increase in OS with chemotherapy or targeted therapy combined with ICI was independent of age, but there was no significant difference in the benefits associated with ICI plus other ICIs (HR = 0.91, 95% CI 0.72–1.16) or radiotherapy (HR = 0.76, 95% CI 0.55–1.06) in the older patients. Both younger and older patients could benefit from ICI combined with chemotherapy and other ICIs, while older patients exhibited no statistically significant benefit from ICI combined with radiotherapy in terms of PFS (HR = 0.74, 95% CI 0.54–1.01).

### Sensitivity Analysis

We performed a sensitivity analysis to detect the influence of a single study on the overall results (Supplementary Figure 9). The absence of each study did not significantly change the overall estimates, which verified the stability of our meta-analysis.

## Discussion

Age affects host immunity and therefore could affect the effectiveness of ICI treatment. Although some meta-analyses have found that the therapeutic effect of ICI was independent of age (21, 22, 43–45), these meta-analyses mainly focused on ICI monotherapy or total cancer treatment and did not specifically study the influence of age on ICI combined with other treatments in the context of NSCLC. To the best of our knowledge, this is the first meta-analysis to investigate the impact of age on the efficacy of ICI-based combination therapy vs. non-ICI therapy in NSCLC patients.

In younger patients, ICI-based combination therapy could decrease the risk of progression by 45% and the risk of death by 33%. In older patients, ICI-based combination therapy could decrease the risk of progression by 36% and the risk of death by 21%. The pooled ratio of HRs for PFS and OS reported in older patients vs. those in younger patients were 1.16 (95% CI, 0.99–1.34) and 1.15 (95% CI, 0.91–1.46), respectively. The differences in the benefits of combination therapy between older and younger NSCLC patients was not statistically significant, indicating comparable efficacy of ICI-based combination therapy in younger and older NSCLC patients.

A meta-analysis also confirmed that both older and younger people can obtain similar benefits from ICI monotherapy (23). Moreover, Allison SB (46) conducted a retrospective study involving 254 participants with metastatic melanoma to confirm this phenomenon. Bora Y (47) identified 1,256 NSCLC patients aged ≥65 years who received nivolumab or pembrolizumab in the Epidemiology, Surveillance and End Results–Medicare linked database, and found no differences in prognosis between different age groups. However, a recent meta-analysis by Wu (45) showed that patients ≥65 years can benefit more from immunotherapy than younger patients. However, the criteria for patients included in this analysis were not strict, including not only NSCLC patients but also renal cell carcinoma patients, melanoma patients, and urothelial cancer patients. The control group included patients receiving first-line chemotherapy, ICI, and only placebo. The above factors may increase the influence of confounding factors on the conclusion.

In our subgroup analysis, younger and older NSCLC patients had a decreased risk of death when treating with PD-1/PD-L1 antibodies plus other therapies, which was consistent with our main results. However, we found that younger patients can obtain greater improvement in PFS than older patients treated with PD-1 inhibitor-based combination therapy. The reason for this phenomenon may be that the naïve T cells generated by the thymus and intratumoral infiltration of regulatory T cells are reduced with age, inhibiting the activation of the immune response (48, 49). We also found that the effect of age on combination therapy compared with its effect on non-ICI therapy was influenced by treatment strategy. The addition of ICI to chemotherapy and targeted therapy can prolong the survival of younger and older people, but older people cannot benefit from ICI combined with radiotherapy in terms of OS. The failure of ICI combined with radiotherapy to enhance OS might be due to the limited number of trials; therefore, more RCTs exploring ICI combined with radiotherapy are needed to investigate the effect of age on efficacy.

Although the data from our included trials met the criteria of the PRISMA statement (33), our meta-analysis has potential limitations as follows. First, the analysis depended on published data, not individual participants' results, moreover, in the trials by Jotte (8), Socinski (7), Hellmann (41), and Govindan (11), the HRs for OS in the 65 years or older group were estimated by calculating the HR of the 65–75 years group and the HR of 75 years or older group using a random-effects model. Second, our meta-analysis was conducted at the study level, and all patients in the clinical trials were in good physical condition, and therefore may not represent the older population in the real-world. Third, significant heterogeneity was found between the included studies, which may be due to the limited number of included trials and different types of ICIs and treatment strategies in our meta-analysis. To minimize the influence of heterogeneity, we used a random-effects model, and performed the subgroup and sensitivity analysis to detect the source of heterogeneity. We cannot exclude other confounding factors that may affect the efficacy of treatment, such as PD-L1 expression, sex, ECOG, and smoking status.

## Conclusion

In conclusion, the meta-analysis showed that ICI-based combination therapy significantly improved OS and PFS in both younger and older patients compared with the effects of non-ICI therapy according to a cut-off age of 65 years in NSCLC patients, but the magnitude of the benefit was independent of age. We also found that treatment efficacy may differ according to the type of ICI and treatment strategy. Therefore, the mechanism of how age affects combination therapy also needs to be further explored and confirmed. Furthermore, we recommend that older people receive combination ICI therapies in the real-world. We also encourage older people to join new and ongoing clinical trials to address this lack of data and geriatric clinical studies.

## Data Availability Statement

All datasets generated for this study are included in the article/Supplementary Material.

## Author Contributions

XY and XT contributed to data extraction, data analysis, and drafting of the manuscript. WH and ZW contributed to study design, data extraction, data interpretation, and data analysis. All authors have access to data in the meta-analysis, are responsible for the integrity of data, the accuracy of the data analysis, and contributed to the critical revision of the manuscript.

## Conflict of Interest

The authors declare that the research was conducted in the absence of any commercial or financial relationships that could be construed as a potential conflict of interest.
