# Supplementary material for: Impact of Age on the Efficacy of Immune Checkpoint Inhibitor-Based Combination Therapy for Non-small-Cell Lung Cancer: A Systematic Review and Meta-Analysis
Source: Front Oncol. 2020 Sep 23;10:1671. doi: 10.3389/fonc.2020.01671 (PMC7538697; doi:10.3389/fonc.2020.01671)

Supplementary Material

Supplementary Table 1: Search strategies for PubMed, EMBASE, and Cochrane database

PubMed		
#1	(non-small cell lung cancer[MeSH]) OR (non-small cell lung cancer)	77304
#2	(((immune checkpoints) OR ctla-4) OR ((((((PD-1) OR PD-L1) OR PD-1 inhibitors) OR PD-L1 inhibitors) OR CTLA-4 inhibitors) OR immune checkpoint)))	35038
#3	(((((((randomized controlled trail) OR controlled clinical trail) OR randomized) OR randomly) OR trial))	2247347
#4	("Drug Therapy"[Mesh]) OR chemotherapy OR radiotherapy OR targeted therapy	3,866,244
#5	#1 AND #2 AND #3AND #4	Final search 980
EMBASE		
#1	'non-small cell lung cancer'	13130
#2	('immune'/exp OR immune) AND checkpoints OR 'ctla 4'/exp OR 'ctla 4' OR (((((('pd l1' OR 'pd 1'/exp OR 'pd 1') AND ('inhibitors'/exp OR inhibitors) OR 'pd l1') AND ('inhibitors'/exp OR inhibitors) OR 'ctla 4'/exp OR 'ctla 4') AND ('inhibitors'/exp OR inhibitors) OR 'immune'/exp OR immune) AND checkpoint)	49401
#3	randomized AND controlled AND ('trial'/exp OR trial) OR (controlled AND trial, AND randomized;) OR 'randomized controlled trial'/exp OR 'randomized controlled trial' OR (pragmatic AND ('clinical'/exp OR clinical) AND trials) OR (randomised AND controlled AND ('study'/exp OR study)) OR (randomised AND controlled AND ('trial'/exp OR trial)) OR (randomized AND controlled AND study;) OR (trial, AND randomized AND controlled)	961293
#4	'chemotherapy'/exp OR chemotherapy OR ' radiotherapy '/exp OR radiotherapy OR ' targeted '/exp OR targeted	1485221
#5	#1 AND #2 AND #3AND#4	Final search 145
Cochrane		
#1	(non-small cell lung cancer[MeSH]) OR (non-small cell lung cancer)	12615
#2	(((immune AND checkpoints) OR ctla-4) OR (((((pd-1) OR pd-l1) OR pd-1 AND inhibitors) OR pd-l1 AND inhibitors) OR ctla-4 AND inhibitors) OR immune AND checkpoint))	3346
#3	chemotherapy OR radiotherapy OR targeted therapy	127859
#4	#1 AND #2 AND #3 Filters：Trails	Final search 668

Supplementary Table 2. Quality assessment: risk of bias by Cochrane Collaboration's tool.

	Study ID	Sequence generation	Allocation concealment	Blinding	Incomplete outcome data	Selective reporting	Other source of bias
West et al (2019)	IMpower 130	Low Risk	Low Risk	High Risk	Low Risk	Low Risk	
Hellmann et al (2019)	CheckMate 227b	Low Risk	Low Risk	Unclear Risk	Low Risk	Low Risk	
Jotte et al (2018)	IMpower 131	Low Risk	Low Risk	Unclear Risk	Unclear Risk	Low Risk	Data from the abstract and the presentation slides
Papadimitrako-poulou et al (2018)	IMpower 132	Low Risk	Low Risk	Unclear Risk	Unclear Risk	Low Risk	Data from the abstract and the presentation slides
Gandhi et al (2018)	KEYNOTE-189	Low Risk	Low Risk	Low Risk	Low Risk	Low Risk	
Paz-Ares et al (2018)	KEYNOTE-407	Low Risk	Low Risk	Low Risk	Low Risk	Low Risk	
Hellmann et al (2018)	CheckMate 227a	Low Risk	Low Risk	Unclear Risk	Low Risk	Low Risk	
Antonia et al. (2018)	PACIFIC	Low Risk	High Risk	Low Risk	Low Risk	Low Risk	
Socinski et al (2018)	IMpower 150	Low Risk	Low Risk	Low Risk	Low Risk	Low Risk	
Govindan et al (2017)	CA184-104	Low Risk	Low Risk	Low Risk	Low Risk	Low Risk	

Supplementary Figure 1: Subgroup analysis: Forest plots of HRs in subgroup analysis stratified by type of ICIs for OS in younger (A) and older (B) patients.

(A)

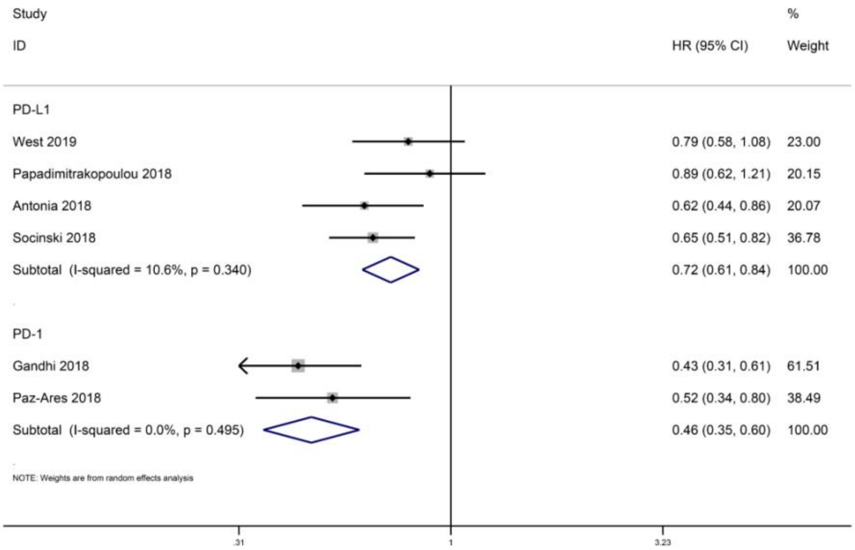

(B)

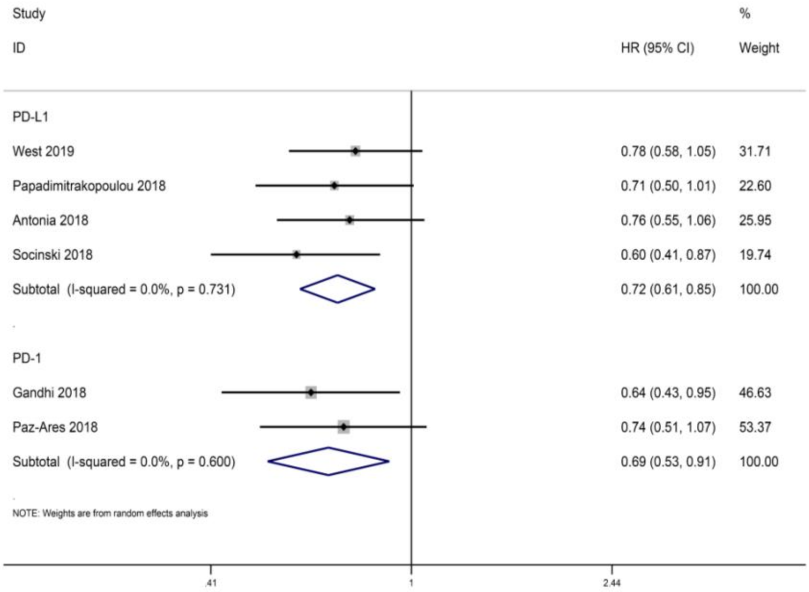

Supplementary Figure 2: Subgroup analysis: Forest plots of the ratio of HRs in subgroup analysis stratified by type of ICIs in older patients to younger patients for OS.

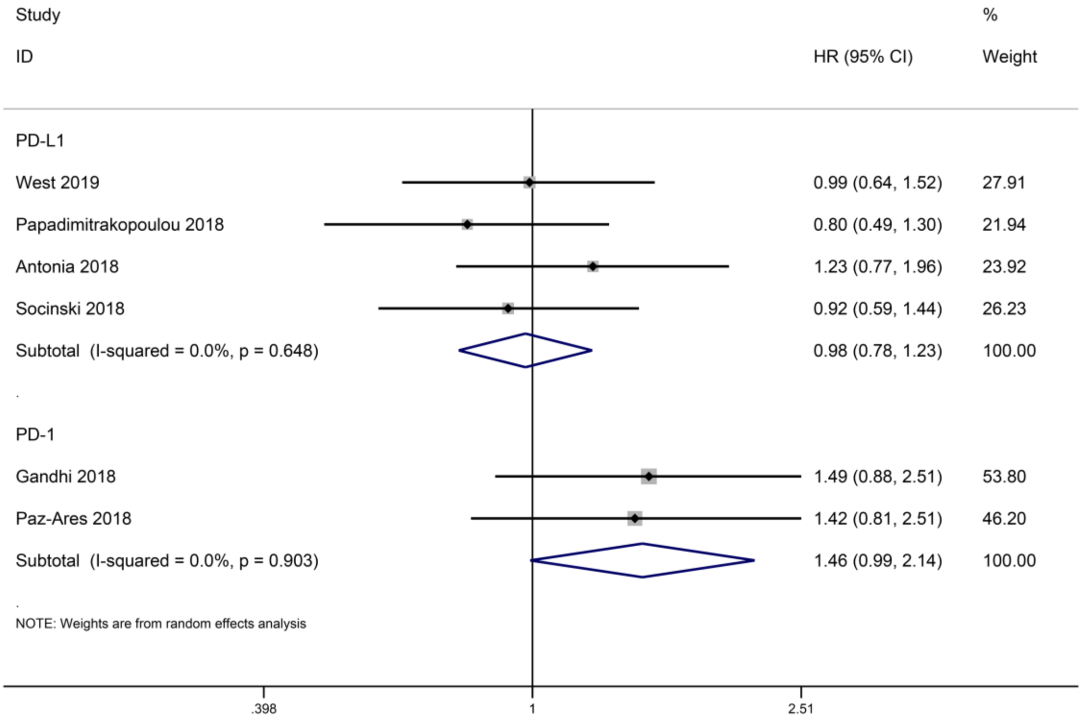

Supplementary Figure 3: Subgroup analysis: Forest plots of HRs in subgroup analysis stratified by type of ICIs for PFS in younger (A) and older (B) patients.

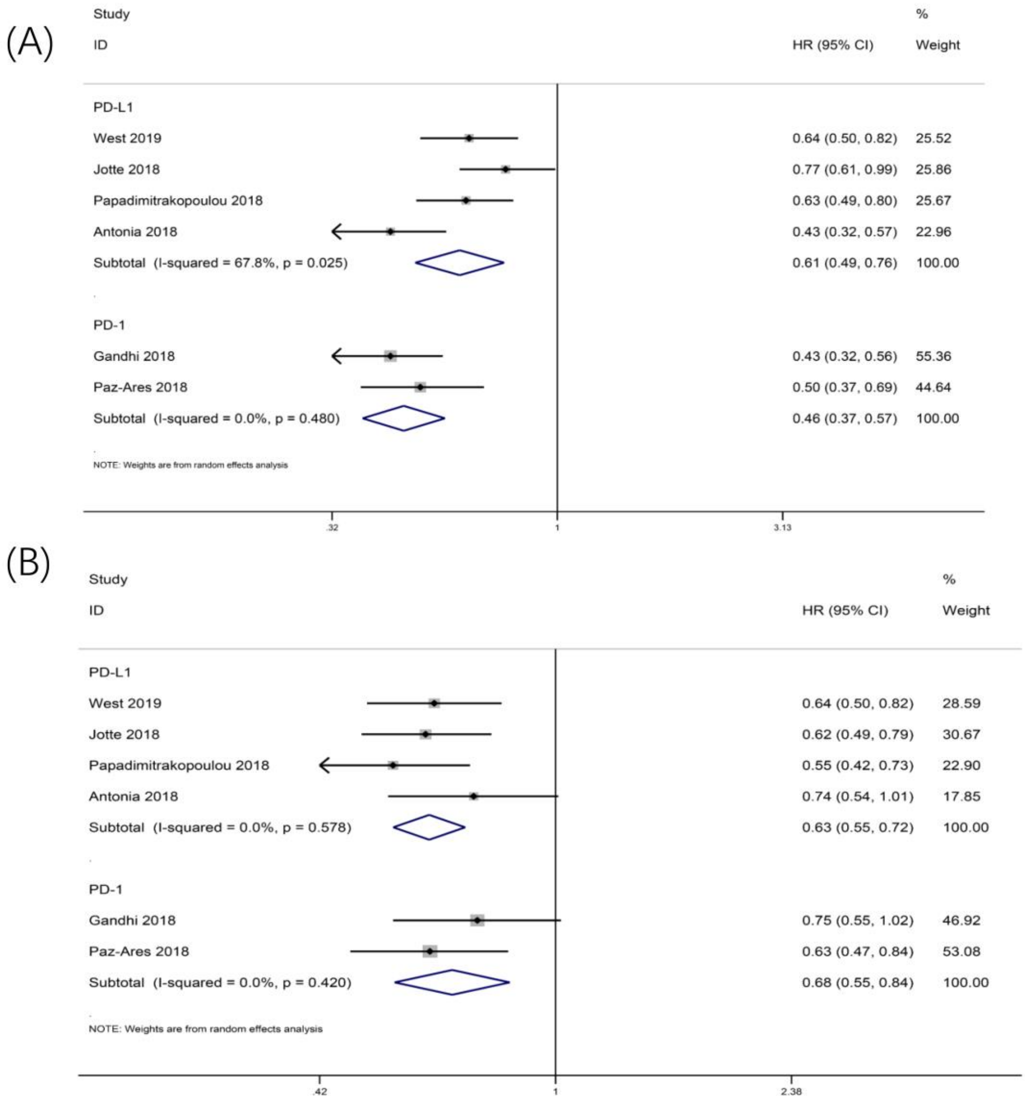

Supplementary Figure 4: Subgroup analysis: Forest plots of the ratio of HRs in subgroup analysis stratified by type of ICIs in older patients to younger patients for PFS.

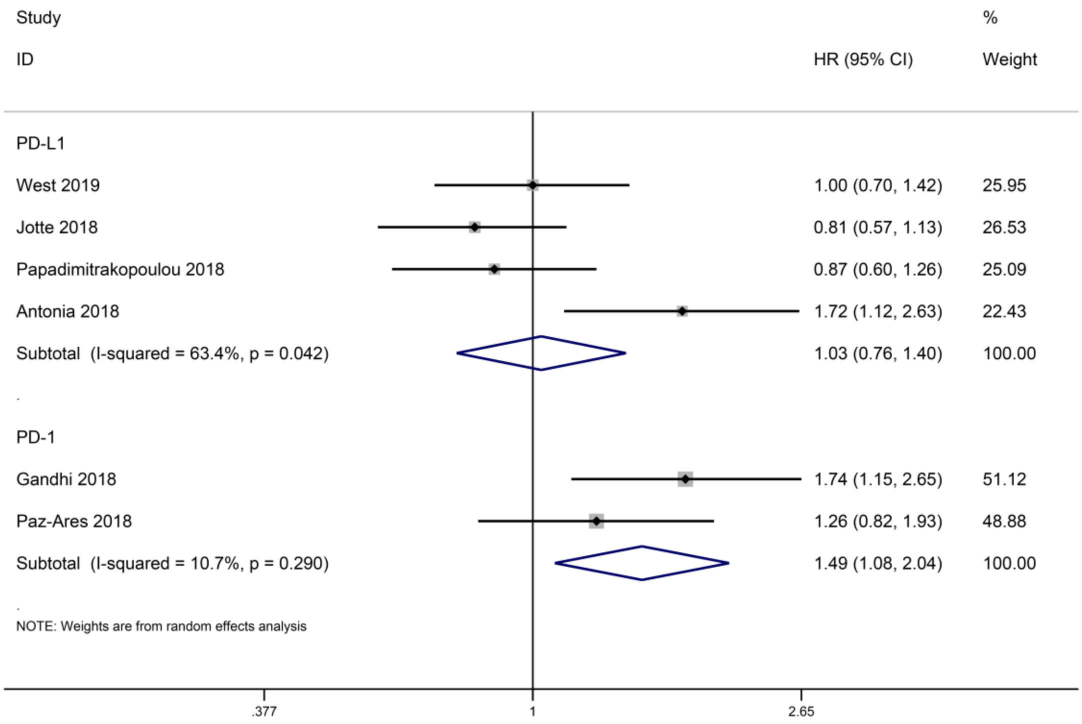

Supplementary Figure 5: Subgroup analysis: Forest plots of HRs in subgroup analysis stratified by treatment strategy for OS in younger (A) and older (B) patients.

(A)

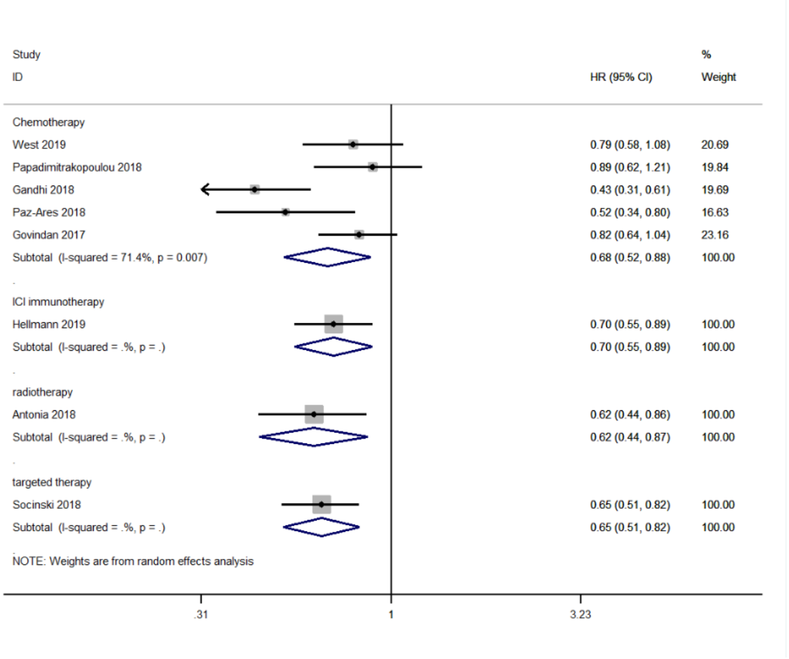

(B)

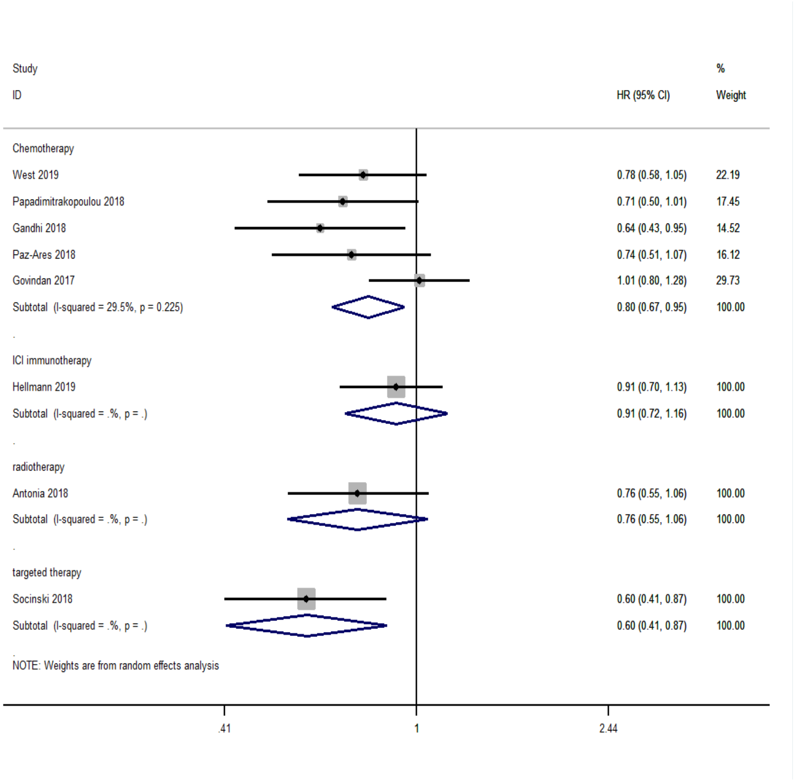

Supplementary Figure 6: Subgroup analysis: Forest plots of the ratio of HRs in subgroup analysis stratified by treatment strategy in older patients to younger patients for OS.

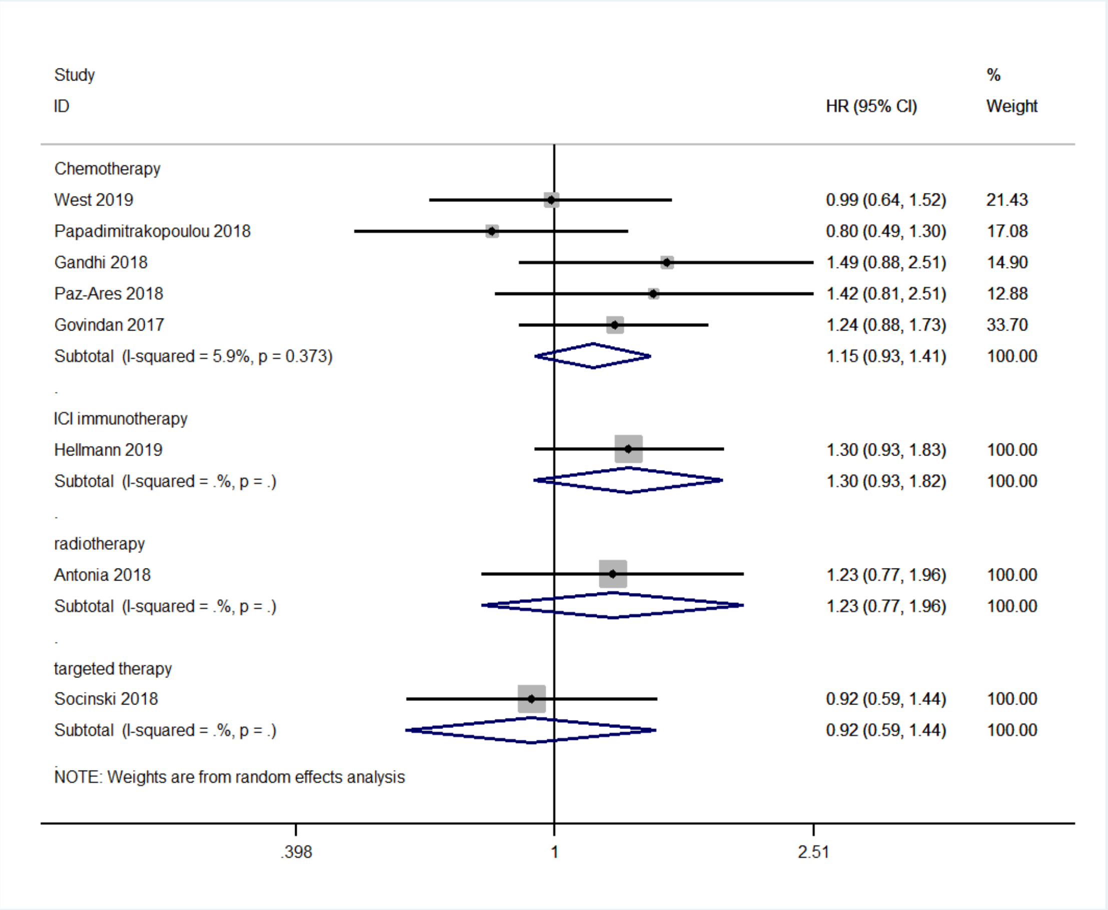

Supplementary Figure 7: Subgroup analysis: Forest plots of HRs in subgroup analysis stratified by treatment strategy for PFS in younger (A) and older (B) patients.

(A)

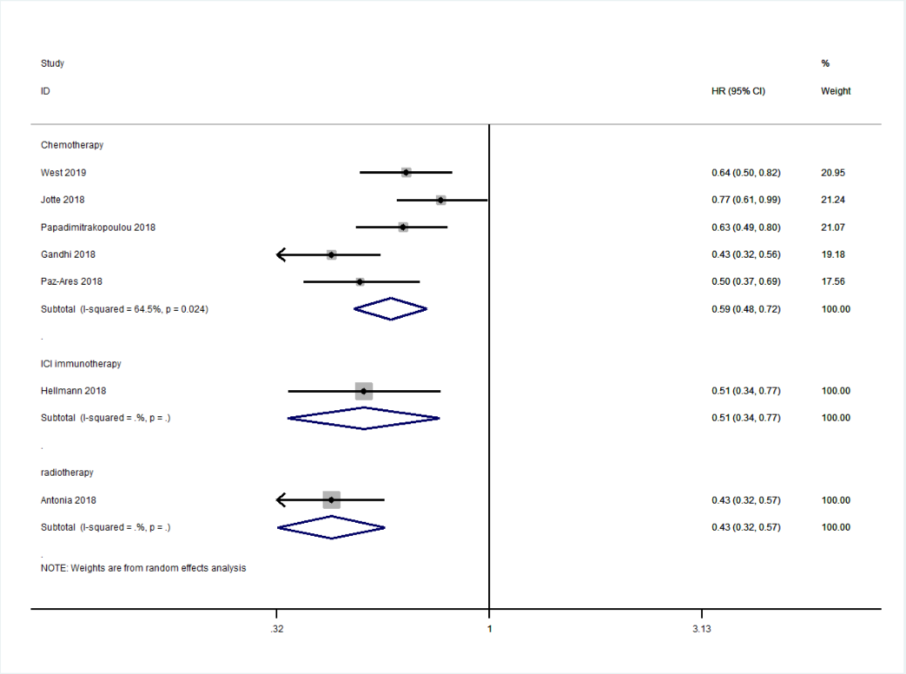

(B)

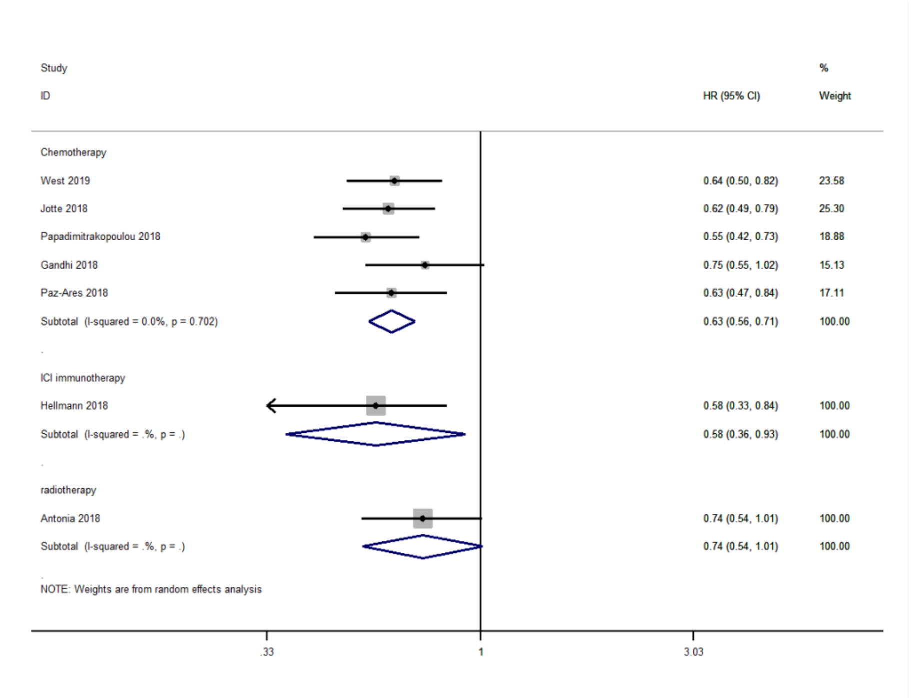

Supplementary Figure 8: Subgroup analysis: Forest plots of the ratio of HRs in subgroup analysis stratified by treatment strategy in older patients to younger patients for PFS.

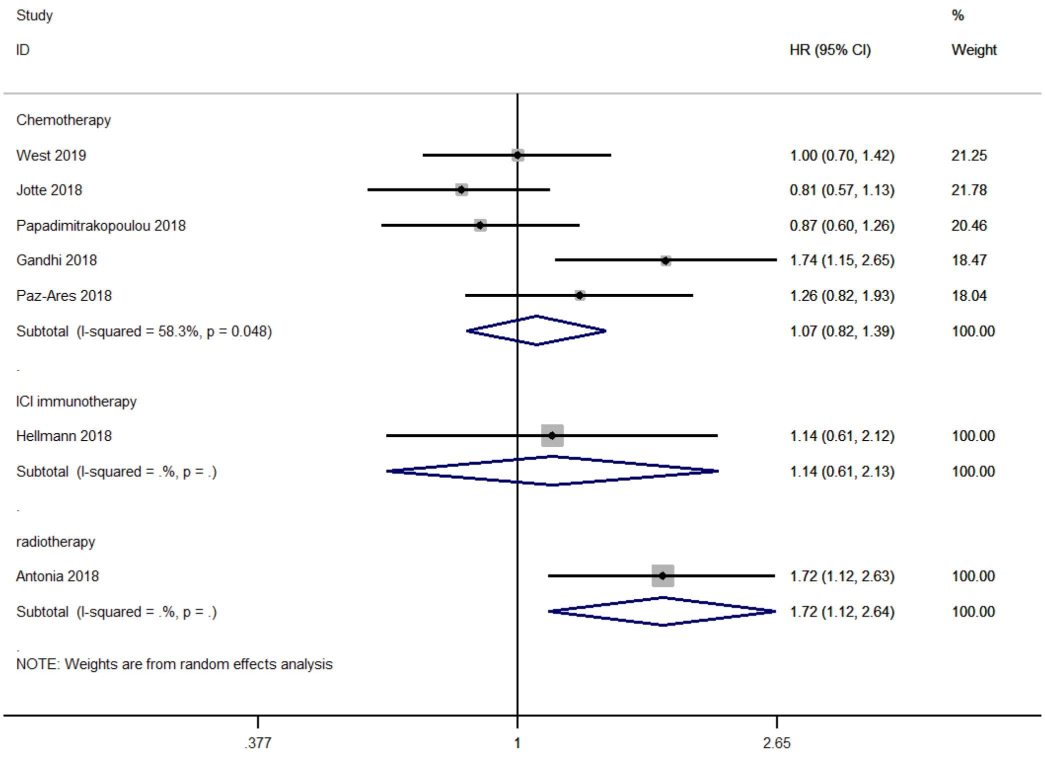

Supplementary Figure 9: Sensitivity analysis: Sensitivity analysis of OS (A) and PFS (B) for younger patients and sensitivity analysis of OS (C) and PFS (D) for older patients in included RCTs.

(A)

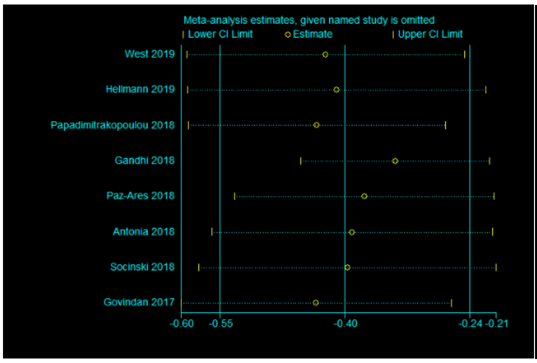

(B)

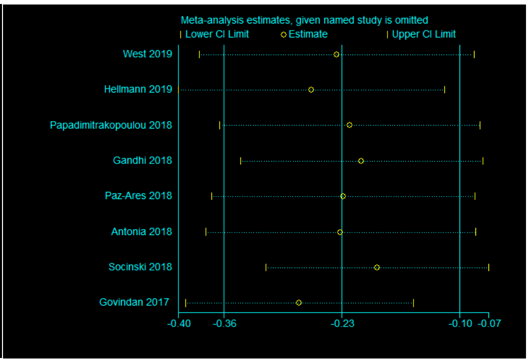

(C)

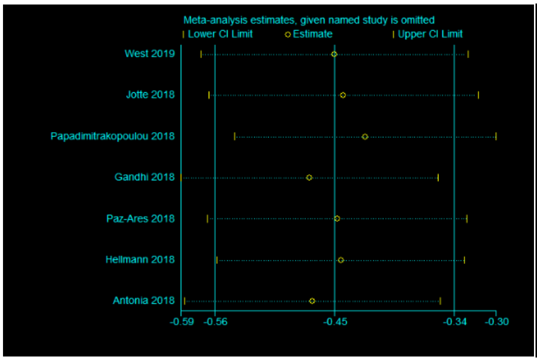

(D)

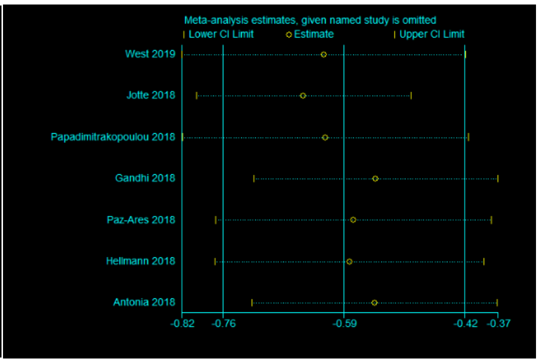

Supplement: Supplementary file 2 [file Data_Sheet_2.PDF]
